# Risky or protective? Online social support’s impact on NSSI amongst Chinese youth experiencing stressful life events

**DOI:** 10.1186/s12888-022-04399-9

**Published:** 2022-12-12

**Authors:** Moye Xin, Lijin Zhang, Chengxi Yang, Xueyan Yang, Meiqiu Xiang

**Affiliations:** 1 School of Psychology, Shaanxi Normal University, No. 199, South Chang’an Road, Xi’an, 710062 Shaanxi China; 2 Shaanxi Key Research Center for Children Mental and Behavioral Health, Shaanxi Normal University, No. 199, South Chang’an Road, Xi’an, 710062 Shaanxi China; 3 Shaanxi Key Laboratory of Behavior and Cognitive Neuroscience, Shaanxi Normal University, Xi’an, Shaanxi, China; 4 College of Liberal Arts, Yulin University, No. 51, Chongwen Road, Yulin, 719000 China; 5 The Institute for Population and Develoment Studies, Xi’an Jiaotong University, Xi’an, No. 28 Xianning West Road, Xi’an, 710049 China

**Keywords:** Stressful life events, Online social support, NSSI, Youth, Gender difference

## Abstract

**Background:**

This study was designed to investigate potential gender differences in the interrelations between different types of stressful life events and non-suicidal self injury (NSSI) among Chinese youth, as well as to test the direct and moderating impacts of online social support on Chinese students’ NSSI engagement under the pressure of different types of stressful life events.

**Methods:**

Based on the data of 2200 students from middle - highschools and universities in Northwestern China, gender difference (male/female binary) in stressful life events, online social support, NSSI and their correlations were analyzed in the study.

**Results:**

Among different types of stressful life events, male students were reported to experience a significantly higher impact of punishment and interpersonal relationship than females. Female students only experienced significantly higher learning pressure than males; Gender difference was not indentified in NSSI among youth; Stressful life events related to punishment could significantly predict NSSI engagement among males. Stressful life events related to learning pressures, interpersonal relationships, and adaption were significantly correlated to NSSI engagement among females; Online social support didn’t had a significant direct effect on youth’s NSSI, although it did significantly moderate the relationship between specific types of stressful life events (i.e., loss, interpersonal relationships, adaption among males and all types among females) and their NSSI.

**Conclusion:**

The present study has provided evidence of specified types of stressful life events being risk factors in affecting youth’s NSSI: For male students, the higher impacts of stressful life events related to punishment they experienced, the more likely they were about to engage in NSSI. For female students, stressful life events related to learning pressure, interpersonal relationships and adaption were all proved as significant predictors and risky factors of female youth’s NSSI; Online social support did not impact on individual’s NSSI engagement directly, but moderated it significantly as a protective factor.

## Introduction

Non-suicidal self injury (NSSI) is defined as “the direct, deliberate destruction of one’s own body tissue in the absence of suicidal intent” [1]. Over the past 30 years, the field of NSSI research has focused primarily on Caucasian Western samples under specific cultural backgrounds [2–4]. The prevalence, characteristics, risk factors, and mechanism of NSSI among have been exclusively studied in countries where a majority of the population is Caucasian [5, 6]. Especially with lifetime prevalence rate of 14–15% among youth (defined by the World Health Organization as person between the ages of 10 and 24, including adolescents and young adults) [7, 8], could be recognized as the high risk group of NSSI.

### The prevalence of NSSI among youth in China

NSSI related studies from non-Western cultures have begun to emerge after 2010 [9–11]. Given the common emotional susceptibility and vulnerability of adolescence, the prevalence and after-effects of NSSI have become equally serious in China [12, 13]. One notable study where 960 Chinese university students (M_*age*_ = 18.69; 66.4% female) from different majors were asked to complete a questionnaire to assess NSSI, which reported that NSSI occured at a higher prevalence (35.2 and 20.4% of male and female students’ lifetime NSSI prevalence) and an earlier onset (the eldest being 24 and the youngest being 15) compared to Western studies [14]. The study of NSSI and its associated factors could be efficient to focus on at-risk groups before taking effective prevention and intervention measures. Sociologists have repeatedly emphasized that research cannot separate individuals from the social environment in which suicide occurs [15, 16], specifically, social psychologists have taken a more comprehensive approach to self-injury research that takes the socio-cultural environment of specific historical periods (e.g., post-COVID era) into further discussion, which might have far-reaching impacts on NSSI [17–19]. Therefore, we are trying to investigate whether different types of stressful life events and forms of social support that youth are experiencing in the current socio-cultural context might have unique impacts on their NSSI or not.

### Stressful life events and NSSI

Stressful life events were not only reported to be critical factors affecting one’s physical and mental health, which referred to the changes and stimulations in individuals’ family, work and/or study environments, that might result in negative psychological and physiological consequences [20], but also could influence one’s engagement in NSSI [21]. Relevant studies have reported that negative outcomes brought by stressful life events played a unique role throughout the internal accumulation of NSSI [22]. Baetens identified that individuals who often experienced great pressure in their daily life were more likely to report NSSI [23]. Studies on students ranging from 14 to 21 years old indicated that stressful life events were significantly correlated to individuals’ NSSI [24, 25]. Individuals who had histories of engaging in NSSI were constantly facing external stimulations which might bring psychological distress and unadaptabilities, treating NSSI as a way of reducing and alleviating stress, claiming that “I intentionally hurt myself to feel better”, which might be useful in the short run [26, 27]. What’s worse, individuals with higher prevalence of NSSI even reported engaging in suicidal behaviors afterwards [28].

### Online social support and NSSI

After the new millennium, internet-based social communication has grown in popularity, which has been used for exchanges of social support, through emotional online communication which might foster a sense of belonging [29]. The rapid development of technology has led to an advancement in possibilities for more diverse communications, which might affect one’s levels of perceived social support [30]. The present study defines online social support as the sense of identity and belonging obtained when one feels need to be understood and respected in the context of emotional exchanges conducted through virtual platforms.

Online social support was proved to be helpful improving the health of individuals troubled by stressful events, but also have implications for the prevention of life-threatening behaviors such as self-injury and suicide [31, 32]. Scholars reported improved quality of life, online social support, and physical health symptoms among participants who engaged in online support groups by posting, replying to messages, and searching for information [33]. Seeking social support from online might generate more positive emotions, reduce stress from challenging life circumstances, thereby decrese the likelihood of resorting to health risk behaviors.

However, some studies suggesting that the impact of online social support on mental health might not always be positive [34–36]. Specifically, given the potentially transient nature of online support, the sense of respect and support brought by virtual communications might not reduce life pressures in a sustained manner [37]. Repeated reliance on online support groups even might lead to Internet addiction such that online social relationships could appear as substitutes for in-person social relations, particularly when individuals did not feel recognized or satisfied in their day-to-day life [38]. Online social groups were also found to bring other hidden dangers, such as exposure to social support from antisocial personality - based online interactive groups, which might breed health risk behaviors in those online interactions [39]. Therefore, the impact of online social support on the relationship between stressful life events and NSSI among youth could be protective or not requires further investigation and clarification.

### Research gaps

#### Research gap 1

The above findings suggested that there might be a close correlation exsiting between NSSI and stressful life events. Although we might infer from previous studies that stressful life events would likely increase, to some extent, the potential for NSSI engagement among youth, the impacting mechanism and specific source of stressful life events should also be taken into consideration, given that different types of stressful life events might differentially impact NSSI among youth.

Due to the rapid growth of stressful life events and NSSI related international psychological and sociological studie s[40–42], emphasizing the significance of gender diversity and its related studies under the multifarious aspects of socio-cultural background, which helped to explain gender bias [43]. Meanwhile, gendered research in China has begun to emerge and uprise as a matter of globalization [44], yet relevant fields of study in China haven’t paid enough attention on gender differences in the context of these variables discussed above, potential gender differences in the impact of different types of stressful life events on NSSI remain largely uninvestigated in China.

Considering the context of global COVID-19 pandemic still influencing, potential stressors such as stressful life events might act as risk factors which could somewhat catalyze the exacerbation of existing anxieties and predict the prevalence of male and female youth’s NSSI psychosocially [17, 34]. The exact mechanism of this relationship and how different types of stressful life events could affect NSSI remain unclear.

#### Research gap 2

When referring to online social support, insufficient literature could be found in related fields in China, only a few studies focused on descriptive analysis on young students of different genders, proving that there was no significant gender difference between the acquisition of online social support (i.e., technical support, emotional support), while male students were proved to have more Internet using motivation [45, 46]. Other empirical studies focused on the impact of online socials support on psychological variables (e.g., internet addiction disorder, pathological internet use, sense of Internet security, etc) among young students, mainly investigating the negative effect of the Internet and its support, without sufficient discussion on gender differences [47–49]. Moreover, related Chinese studies have rarely assessed the relationship between stressful life events, NSSI, and online social support among youth. Thus there could be a lack of empirical research on the potential direct or indirect impact test of online social support on the relationship between stressful life events and NSSI among youth, specifically, under the background of global epidemic still lingering [34], whether online social support could act as one long - term and effective support source considering social distance and physiological safety, which requires further investigation.

### Current study

According to research gap 1, which aimed at testing whether different types of stressful life events could become stress factors affecting youth’ NSSI and indicating whether there were gender differences in the process of impact of different types of stressful life events on their NSSI.

According to research gap 2, it remained to be determined whether online social support could play a stress-buffering role on NSSI among youth. Even if we presume that online social support could impact the relationship between stressful life events and NSSI among youth significantly, this impact could be protective or not remained uninvestigated.

**Fig. 1:**
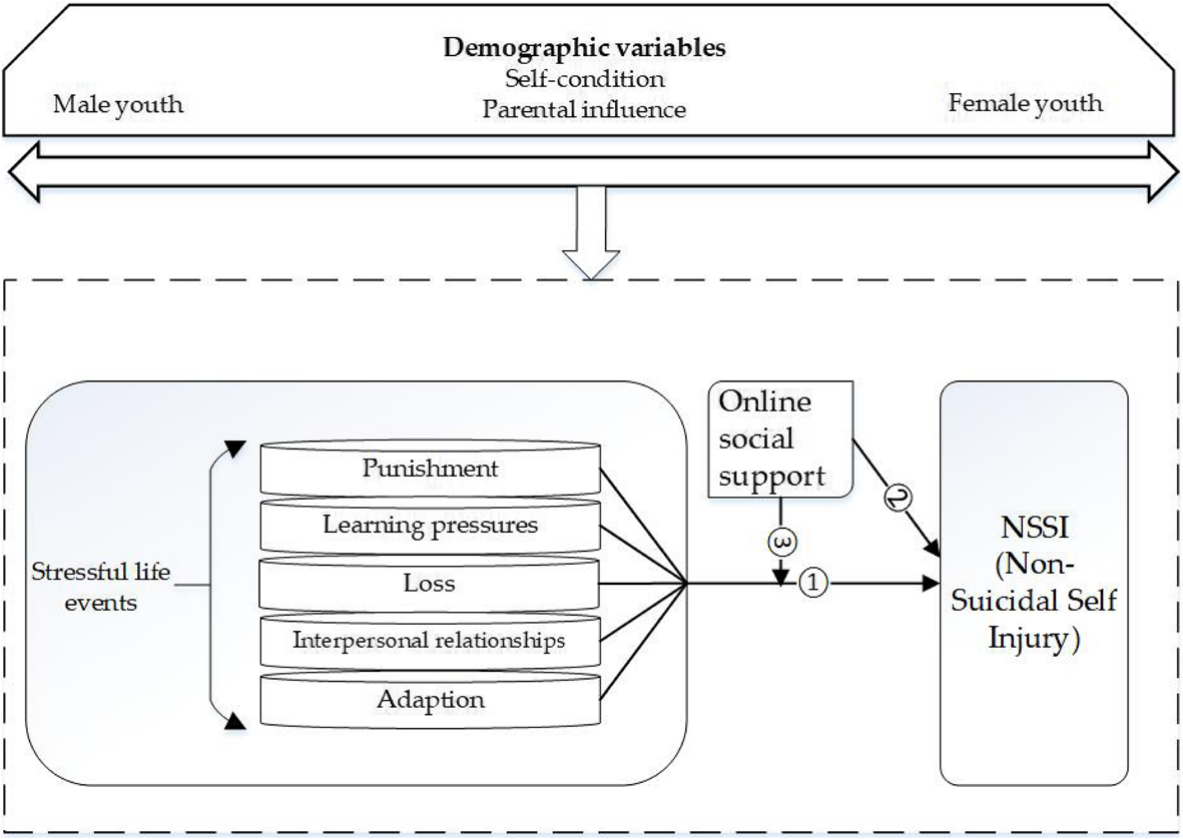
The direct and moderating effects of online social support on NSSI (Path 2–3) among youth under the pressure of stressful life events (Path 1)

The present study thus assessed four main research objectives. The first two objectives were proposed corresponding to research gap 1. Objective 1 was (1) to assess potential differences in (a) NSSI, (b) overall reports of stressful life events, and specific types of them (i.e., punishment, learning pressures, loss, interpersonal relationships, adaption), (c) online social support among Chinese middle-high school and university students; Objective 2 was (2) to determine the potential role of different types of stressful life events in associating with NSSI to determine whether specific types of stressful life events had impacted during the process of NSSI engagement among youth (Fig. 1, Path 1).

The last two objectives were proposed corresponding to research gap 2. Objective 3 was to determine whether online social support had direct effects on NSSI among youth (Fig. 1, Path 2). Objective 4 was to determine whether online social support could significantly moderate the relationship between stressful life events and NSSI among youth (Fig. 1, Path 3).

4 hypotheses were therefore listed accordingly as follows:

H1A: There would be gender differences in overall stats and specific types of stressful life events, male students might experience more stressful life events than females.

H1B: There would be gender differences in online social support among youth, male students might receive more online social support than females.

H1C: There would be gender differences in NSSI engagement among youth, male students might engage in more NSSI behaviours comparing to females.

H2: The potential role of specific types of stressful life events would increase NSSI engagement among youth proving they could be risky.

H3: Online social support would have significant direct impact on NSSI engagement among youth.

H4: Online social support was hypothesized to significantly moderate the relationship between stressful life events and NSSI engagement among youth indicating it’s protective.

## Materials and methods

### Participants

Participants were a sample of 2, 200 students (998 males, 1202 females; 1073 middle - high school students, 1127 university students; *M*_age_ = 17.8 years; age range: 11–24 years) recruited from three middle - high schools and two universities in Northwestern China. The stratified proportion sampling methods were adopted for selecting male and female students from grades 7 to 11 of three middle - high schools and freshman to junior of two universities during the survey. Among middle - high school students, 136 students were from grade 7, 12.7%; 248 students from grade 8, 23.1%; 260 students from grade 9, 24.2%; 218 students from grade 10, 20.3%; and 211 students from grade 11, 19.7%. Among university students, 386 students were freshmen, 34.3%; 411 students were sophomores, 36.5%; and 330 students were juniors, 29.3%. Parents or legal guardians gave permission for students’ participation (under 18) by signing consent forms before the survey.

### Measures

#### NSSI engagement

In order to assess youth’s engagement in NSSI, the Non-suicidal Self-Injury Assessment Tool (NSSI-AT) [5], was selected and translated into Chinese. By asking, “*have you ever engaged in these behaviors below that you didn’t intentionally harm yourself for the purpose of suicide*?”, measured as “*0 = no, 1 = yes*”. Based on the 14 kinds of NSSI behaviors stipulated by NSSI-AT, this study refers to the relevant research of youth in the Chinese social and cultural environment, youth tend to engage in different methods of self-injury from Western samples [12], including “1. *Swallowing items that cannot be digested, such as plastic, stone, etc; 2. Taking or swallowing too much medicine (beyond the medical advice); 3. Burn yourself with cigarette butts* “; *4. Carve your arms,thighs or other body parts to make a “blood tatoo”.* After adding the above 4 kinds of specific NSSI behaviors, a total of 18 specific NSSI behaviors was defined. The internal consistency of this scale was found to be adequate for the present study (α = .82).

#### Stressful life events

The Adolescent self-rating stressful life events check-list (ASSLEC) was used to examine stressful life events [50]. This scale consisted of 27 items, each categorized within 1 of 6 types of stressful life events: interpersonal relationships (e.g., *was misunderstood or wronged; was discriminated against or treated coldly*), learning pressures (e.g., *failure in an exam*), punishment (e.g., *was criticized or punished at school*), loss (e.g., *sudden death of relative or friend; experienced theft or lost items*), adaption (e.g., *transfer or suspension, major changes in daily routines*).

The original scale was adapted for the purposes of the present study to accommodate participants’ age and better distinguish the relative impacts of stressful life events. Relevant factors were extracted by means of a principal component analysis and then rotated using the maximum variance method. A total of 5 factors were extracted; this 5-factor model met the numerical requirements of the fitting index test without changing the original 6-factor connotation of the original scale, and the fitting index result was more ideal than the 6-factor model. The correlation coefficient of the 5-factor model was 0.694 ~ 0.788 (*p < .01*) and the structure validity was improved. In terms of structure and content, compared with the original 6-factor model, the 5-factor model omits the “other types” factor. In the present study, this scale assessed 5 types of stressful life events among adolescents and young adults: punishment, learning pressures, loss, interpersonal relationships, and adaption. The scale asks respondents to rate each item on a 5-point Likert scale based on the extent to which each event impacted their life, from 1 (*no impact*) to 5 (*extremely severe impact*). The modified ASSLEC indicated great reliability (α = .87).

#### Online social support

The 23 - item online social support scale designed by Liang [51] was adopted to assess the degree to which participants have obtained emotional and practical support through the Internet. Participants were asked to indicate the extent to which they have received online social support using a 5-point Likert scale ranging from 1 (*no*) to 5 (*always*). Sample items include, *“I found more friends through common interests and hobbies on the Internet.”* and “*Comparing to real life communication, I choose to spend more time in online communication, because it’s more reliable and relaxing.*” A mean score is calculated whereby the higher the score, the more online social support received. The scale has reported sufficient reliability (α = .835).

### Data collection

The present study used convenience sampling to recruit students within three middle - high schools and two universities who expressed interest in participating. All questionnaires were anonymous. The survey started after getting all participants’ approval. Each participant were asked to complete a self-applicable physical questionnaire. A total of 2400 questionnaires were distributed and 2200 valid questionnaires were recovered, the participation rate was therefore 91.67%.

### Data analysis strategy

A series of one-way ANOVAs were conducted to analyze potential gender differences in stressful life events and online social support, while Chi-square test was adopted to analyze the gender differences in NSSI engagement amongst youth.

Furthermore, a step-wise binary logistic regression using SPSS 19.0 was adopted to test whether different types of stressful life events (Step 1), and demographic variables (Step 2) were significantly associated with NSSI engagement (Model 1–2) for both genders. Specifically, model 1–2 (M = male; F = female) regarded NSSI status as the dependent variable. Model 1 included stressful life events as independent variables, while model 2 included additional demographic variables (i.e., age, only child or not, parents’ marital status). Please refer to Fig. 1 and Table 1 for details.

**Table 1:** Specific information of related models and steps (hypothesis 2)

Dependent Variables	Models	Independent Variables
whether NSSI occured (reference: No)	Model 1 M (Step 1)	Stressful life events
	Model 2 M (Step 2)	Stressful life events (e.g., punishment) + Demographic variables (i.e., age、only child or not、parents’ marital status)
	Model 1F–2F	Same procedure as above

Taking hypotheses 1 and 2 as research premise of verifying hypotheses 3 and 4, the binary logistic regression method was adopted to test the direct and moderating effects of online social support on NSSI among youth under the pressure of stressful life events, across genders. 6 models were thus assessed, see Table 2 for details. Model 3 (M/F) included online social support (OSS) as an independent variable to measure its direct effect on the relationship between stressful life events (independent variable) and NSSI engagement (dependent variable). Subsequently, on the basis of Model 3, the interaction terms for punishment, learning pressures, loss, interpersonal relationships, and adaption as different types of stressful life events were successively included in Models 4–8 to determine the potential moderating effects of offline and online social support on NSSI.

**Table 2:** Specific information of related models and steps (hypotheses 3–4)

Dependent Variables	Model	Independent Variables
NSSI engagement among males	Model 3 M	Stressful life events + OSS + Demographic variables (Age、only child、parents’ marital status)
	Model 4 M	Stressful life events + OSS + Punishments * OSS + Demographic variables
	Model 5 M	Stressful life events + OSS + Learning pressures * OSS + Demographic variables
	Model 6 M	Stressful life events + OSS + Loss * OSS + Demographic variables
	Model 7 M	Stressful life events + OSS + Interpersonal relationship * OSS + Demographic variables
	Model 8 M	Stressful life events + OSS + Adaption * OSS + Demographic variables
NSSI engagement among females	Model 3F - 8F	Same procedure as above

*Note:* M stands for “males”, F for “females”, OSS for “online social support”

## Results

### Descriptive analysis results

Results from one-way ANOVAs revealed significant gender differences in overall reports of stressful life events (F *=* 8.335*, p < .001*) and online social support (F *= 12.638, p = 3.691*), whereby males all reported higher levels of both comparing to females, but gender difference of online social support was not statistically significant (see Table 3 for details).

**Table 3:** Descriptive analysis results

	Male(***N*** = 998)		Female(***N*** = 1202)	
	Min/Max	Mean (SD)	Min/Max	Mean (SD)
Stressful life events (Overall)	1/5	2.01(0.71)	1/5	1.87(0.61)
	F = 8.335***			
Punishments	1/5	1.72(0.75)	1/5	1.48(0.63)
	F = 7.218***			
Learning pressure	1/5	2.33(0.45)	1/5	2.51(0.79)
	F = 2.834*			
Loss	1/5	1.71(0.59)	1/5	1.61(0.58)
	F = 5.193			
Interpersonal relationship	1/5	2.47(0.76)	1/5	2.33(0.65)
	F = 8.771**			
Adaption	1/5	1.76(0.69)	1/5	1.79(0.58)
	F = 1.547			
Online social support (Overall)	1/5	2.71(0.73)	1/5	2.68(0.65)
	F = 12.638			

Note: +, p < 0.1；*, p < .05；**, p < .01；***, p < .001；

Moreover, analyses of gender differences across 3 specific types (punishment, learning pressure & interpersonal relationship) among 5 total types of stressful life events, revealed that males could have a significantly higher degree of stressful life events related to punishment (F *=* 7.218*, p < .001*), and interpersonal relationship (F *=* 8.771*, p < .01*). Yet females only scored significantly higher than that of males in one specific stressful life events, which is learning pressure (F *=* 2.834*, p < .05*). No significant gender differences emerged in reports of stressful life events related to the category of loss (F = 5.193, *p = 1.603*) and adaption (F = 1.547, *p = 1.368*). Refer to Table 3 for detailed information.

A total of 497 participants reported engaging in NSSI indicating a prevalence rate of 22.6%. Prevalence rates were found to be 21.2 and 23.4% for males and females, respectively. Yet Chi-square test results showed no significant difference in the prevalence of NSSI between male and females (χ^*2*^ = 1.335, *p* = *3.171*).

### Different types of stressful life events’ impact on NSSI

Table 4 reported the results from binary logistic regression on stressful life events associated with the prevalence of NSSI behaviors among youth. For male students, the results of models 1 M and 2 M revealed that only one specific type of stressful life events had significant positive impact on their NSSI: punishment (β = 0.228*, p < .001*). This has indicated that increased reports of stressful life events related to punishment was significantly associated with increased NSSI engagement among males. After the addition of demographic variables, the impact of punishment on male students’ NSSI did not change significantly, but the impact of adaption on NSSI slightly increased (β *=* 0.177*, p < .1*). Parents’ marital status (i.e., remarriage (β *=* 0.186*, p < .1*), divorce (β *=* .162*, p < .05*)) had relatively significant impacts on males’ NSSI engagement.

**Table 4:** Impacts of different types of stressful life events on NSSI

Dependent variable: whether NSSI occured (reference: No)	NSSI for males (***N*** = 998)		NSSI for females(***N*** = 1202)	
**Independent variables:** Stressful life events	Model 1 M	Model 2 M	Model 1F	Model 2F
Punishments	0.228***	0.247***	0.236	0.239
Learning pressure	0.012	0.016	0.251**	0.298***
Loss	0.031	0.033	0.083	0.088
Interpersonal relationship	0.011	0.013	0.153***	0.161***
Adaption	0.141	0.177+	0.037+	0.108*
**Control variables (Demographic)**				
Age		0.051		−0.139**
Studying period: University (Middle-highschool)		0.059		0.013
Only child or not:no (yes)		0.019		−0.011
Parents’ marital status: Remarriage (First marriage)		0.186+		0.241*
Divorced (First marriage)		0.162*		0.117**
Widowed (First marriage)		0.038		0.028
95% CI	0.85, 0.88**	0.86, 0.92**	0.93, 0.97**	0.87, 0.98**
−2 Log Likelihood	586.34***	600.37**	653.18***	793.22***
Cox & Snell R^2^	0.016	0.038	0.012	0.036
Nagelkerke R^2^	0.015	0.037	0.014	0.031

Note: M stands for “males”, F for “females”. All regression coefficients reported are standard. +,p < .1；*, p < .05；**, p < .01；***, p < .001

For female students, models 1F and 2F revealed a different pattern of results Specifically, learning pressure *(*β *=* 0.251*, p < .01*), interpersonal relationships (β *=* 0.153*, p < .001*), and adaption (β *=* 0.037*, p < .1*) all emerged as significant positive predictors of females’ NSSI engagement. This has indicated that increased reports of stressful life events related to learning pressures, interpersonal relationships, and adaption were significantly related to increased NSSI engagement among females. After the addition of demographic variables, the impact of learning pressure and adaption on female students’ NSSI increased (β *=* 0.298*, p < .001*; β *=* 0.108*, p < .05*) while the impact of other stressful life events on NSSI did not change significantly. Only age, parents’ marital status (i.e., remarriage, parental divorce) had significant impacts on females’ NSSI (β *=* 0–.139*, p < .01*; β *=* 0.241*, p < .05*; β *=* 0.117*, p < .01*).

### The direct and moderating effect of online social support

As presented in Table 5 (Model 3 M), results revealed that online social support did not have a significant direct effect on NSSI among male youth (β = .030*, p* = .362). The amount of online social support received by male students was not directly related to the engagement of their NSSI. However, with the addition of interactive variables (Models 4 M - 8 M), it was found that online social support had a significant negative moderating effect on the relationship between 3 types of stressful life events and NSSI among male youths (loss, interpersonal relationships, adaption, β = − 0.385, − 0.381, − 0.386, − 0.384, − 0.388, all *p*’s < .001). In other words, online social support has reduced the impact of those types of stressful life events on male students’ NSSI engagement as a protective factor.

**Table 5:** The direct and moderating effects of online social support on NSSI engagement among male and female youth

Independent Variables whether NSSI occured (reference: No)	NSSI for males (***N*** = 998)						NSSI for females(***N*** = 1202)					
	Model 3 M	Model 4 M	Model 5 M	Model 6 M	Model 7 M	Model 8 M	Model 3F	Model 4F	Model 5F	Model 6F	Model 7F	Model 8F
***stressful life events***												
Punishment	0.247***	0.383***	0.288***	0.341***	0.311***	0.293***	0.239	0.248***	0.041	0.038	0.041	0.059
Learning pressures	0.016	0.019	0.213***	0.011	0.013	0.014	0.298**	0.331**	0.357***	0.325***	0.333***	0.327***
Loss	0.033	0.035	0.038	0.187***	0.002	0.004	0.088	0.093	0.091	0.113***	0.098	0.094
Interpersonal relationships	0.013	0.018	0.016	0.014	0.183***	0.002	0.161***	0.183***	0.186***	0.183***	0.184***	0.184***
adaption	0.177+	0.181	0.187	0.188	0.188	0.293***	0.108	0.066	0.053	0.053	0.047	0.333***
***Social support***												
Online social support	0.030	−0.014	−0.015	− 0.014	−0.158	− 0.017	0.074	0.070	0.337	0.161	0.093	0.164
***Control variables***												
Age	0.051	0.059	0.053	0.056	0.055	0.062	−0.139**	−0.284***	−0.280***	−0.277***	− 0.279***	−0.270***
Schooling stages: University (Middle school)	−0.003	−0.005	− 0.004	−0.003	− 0.001	−0.006	− 0.013	−0.019	− 0.011	−0.013	− 0.011	−0.011
Single child (not single)	−0.019	−0.025	− 0.024	−0.025	− 0.025	−0.024	− 0.011	0.030	0.031	0.039	0.039	0.032
Marital status of parents: Remarriage (first marriage)	0.186+	0.198+	0.198+	0.198+	0.197+	0.199+	0.241*	0.253*	0.253*	0.251*	0.249*	0.249*
Divorced (first marriage)	0.162**	0.183**	0.188**	0.185*	0.184*	0.181*	0.117**	0.120**	0.118**	0.117**	0.114**	0.119**
Widowed (first marriage)	0.038	0.040	0.049	0.040	0.040	0.049	0.028	0.029	0.020	0.021	0.029	0.020
***Moderation analysis***												
Punishment * Online social support		−0.385						−0.415***				
Learning pressures * Online social support			−0.381						− 0.410***			
Loss * Online social support				−0.386***						− 0.400***		
Interpersonal relationships * Online social support					−0.384***						− 0.425***	
adaption * Online social support						−0.388***						− 0.423***
95% CI	0.77, 0.83**	0.73, 0.75**	0.81, 0.86**	0.83, 0.86**	0.85, 0.88**	0.83, 0.86**	0.81, 0.83**	0.78, 0.85**	0.78, 0.86**	0.75, 0.78**	0.81, 0.93**	0.86, 0.89**
−2 Log Likelihood	518.52***	533.78**	568.77***	571.38**	552.66***	563.51**	616.32***	746.58***	724.67***	738.54***	771.38***	767.31***
Cox & Snell R^2^	0.051	0.063	0.067	0.068	0.063	0.061	0.055	0.066	0.062	0.063	0.067	0.068
Nagelkerke R^2^	0.067	0.076	0.072	0.077	0.073	0.074	0.073	0.085	0.086	0.084	0.087	0.087

Note: M stands for “males”, F for “females”. All regression coefficients reported in the table were standard regression coefficients. + p < .10；*p < .05；**p < .01；***p < .001

As presented in Table 5 (Model 3F), results revealed that online social support did not had a significant direct effect on NSSI among female youth as well (.074*, p* = .346).

With the addition of interaction terms (Models 4F–8F), it was found that online social support had a significant moderating effect on the relationship between all types of stressful life events and NSSI among female youth (β = − 0.415, − 0.410, − 0.400, − 0.425, − 0.423, all *p*’s < .001). Online social support also reduced the impact brought by all types of stressful life events on female students’ NSSI as a protective factor, similar to the results of male students.

## Discussion

Significant gender difference was discovered in overall status of stressful life events among youth across genders, male students were found to experience more stressful life events than females as hypothesized (H1A). This is consistent with related study claming that male students were more likely to get exposed to stressful events than females, which could endanger their mental health afterwards [52]; As hypothesized (H1B), significant gender difference was identified in overall reports of online social support among youth across genders, male students received more online social support than females. This is consistent with an existing study indicating that when encountering physical and psychological problems, male students tended to seek help from the Internet, while female students would rather seek help from offline or “real - life” instead [25]; Not as hypothesized (H1C), no significant gender difference was found in NSSI among youth, which is consistent with previous studies relying on Chinese samples [22, 53].

### The impact of different stressful life events on NSSI among young students

However, our results revealed that stressful life events might act as risk factors for youth’s NSSI as hypothesized (H2), regardless of their gender. Specifically, it is noteworthy that specific types of stressful life events (i.e., punishment, adaption) were significantly correlated to male students’ NSSI, while other types of stressful life events had no significant impact on their NSSI. These findings could be interpreted by the “gender stereotype” which is commonplace in Chinese social culture [54, 55]. This stereotype regarded males as exhibiting stronger psychological endurance and aggressiveness than females, who were generally considered to be gentle and mild. This kind of “gender stereotype” or “gender bias” has led to biased perceptions of female students, proving male students in China were found to frequently get exposure to incidents of punishment comparing to females [56], placing them at-risk for experiencing NSSI as a result. Among youth who experienced a long-term separation from family members, males were more likely to report mental health difficulties than females, a finding which was partly explained by their relatively higher levels of introversion and neuroticism [57]. These male students showed less progress relative to females in terms of intellectual development and environmental adaptability over time, and were more likely to report more negative self-evaluations. The above psychological factors were all proved to be risk factors that might lead male students to engage in risky behaviors or NSSI [58]. However, no gender difference was found in NSSI among youth who spent more time living with their families [59].

Among female students, stressful life events related to interpersonal relationships, learning pressures and adaption all had significant impacts on their NSSI. This could possibly be explained by the perception and education of “gender stereotype” or “gender bias” deeply rooted from traditional Eastern- Asian families’ nurturing value and culture over centuries as mentioned above [55]. Although this kind of prejudice or bias is vanishing gradually with the development of social economic and educational level, the afterwave of this specified “gender stereotype” or “gender bias” is still lingering and influencing [60]. Corresponding interventions and countermeasures are strongly needed to prevent NSSI from happening, especially among female students who just encountered or experienced those stressful life events, which is also for better and faster elimination of gender bias.

In particular, for students who experienced negative feedbacks from interpersonal relationships and adaption in campus, supporting system (e.g., mental health education courses, psychological counseling, etc) in each middle-high school and university could be of great help, so that young students in the emotional and rational development period might be possible to have a systematic understanding of their own psychological state, improving the emotional quotient of classmates, avoiding excessive interpersonal conflicts which might raise the psychological pressure level or even NSSI related behaviours afterwards [61, 62]. Furthermore, the information confidentiality and fairness of the supporting system should be enhanced, so that more young people with psychological crisis or confusion could be more willing to release their internal pressure and communicate more proactively when receiving effective emotional counseling, in order to get more timely, customized, professional and private support [63–65]. Meanwhile, the ability to efficiently deal with individual stress, cope with academic setbacks and failures, and correctly express their feelings and thoughts should be taught by college counselors or full-time psychological tutors and aquired by young students with the age growth, in order to better coping with increasing learning pressures with grades [66].

### The direct and moderating effect of online social support

Contrary to our hypothesis (H3), online social support did not directly affect the NSSI engagement of youth (regardless of their gender). No relevant studies in China could be found to support this finding, but a study by Biernesser [67] found that male youth with NSSI reported a preference for offline social support from family, friends, or mental health professionals over online social support. Specifically, participants reported preferring to share their confusion and thoughts around NSSI related topics and conversations in a more direct, face-to-face style. Online communication groups on self - injury issues were more commonly used by individuals in early stages of NSSI or their family members, while discussions centered on understanding each other’s experience, such communications might not serve to prevent or reduce the engagement of NSSI [68]. It could be an proper explaination which claimed that online social support was not an important factor in terms of directly affecting NSSI among young students of different genders.

Meanwhile, online social support had a significant moderating effect on the NSSI of youth (regardless of their gender) under the pressure of stressful life events as hypothesized (H4), which has significantly buffered the impact of 3 specific types of stressful life events (i.e., loss, interpersonal relationships, adaption) on NSSI engagements among male students and all types of stressful life events among female students, indicating itself as a protective factor and effective strategy against various types of stressors and NSSI for both male and female students. This finding is consistent with previous research and might be explained by the privacy and indirect characteristic of online social support. Specifically, with the global epidemic still influencing, young students experiencing NSSI as a result of stressful life events might or had to turn to the Internet for support as they might be hesitant to disclose details of their negative thoughts or emotions in-person due to feelings of shame or guilt, or a desire for a more private and/or confidential social exchange that can be accomplished virtually, or due to pandemic prevention and control [17, 19, 34]. Thus, the private nature of online social support might enable youth to seek help from online forums without restraint. Online support has the potential to buffer the negative impacts of challenging life events they might be experiencing, thereby reducing the impact of these events on their NSSI prevalence [29]. In short, the inherent characteristics of online social support might be optimal for potentially reducing the impacts of stressful life events on NSSI among males, proving it’s protective to mental health among male students.

## Limitations and future directions

The present study has following limitations: 1) The present sample consisted of students attending middle - high schools and universities in provincial capital and prefecture - level cities. As such, samples from county - level and township - level might not be represented within the present study. 2) This study relied only on cross - sectional self - report measures to examine NSSI. Further longitudinal investigations are needed to understand potential contributors to the patterns of engagement in NSSI among Chinese youth for future studies. 3) The method of MANOVA might be a more appropriate analysis to capture interactions as well as reduce family wise error present in multiple analysis. Further studies using mutiple methods (e.g., MANOVA, etc) are needed to investigate potential contributors to the above patterns of mechanism in youth’s NSSI. 4) The reliance on the male/female binary. Compared with the general population, transgender/gender non - conforming (TGNC) youth might have a higher risk of NSSI, which has already aroused the attention of relevant academia internationally [69–72]. Yet this filed of study remains academic blank and constantly being neglected by relative Chinese scholars and researchers. Future studies relying on more samples of gender - diversity could be of great significance to contribute to the related fields.

## Conclusions and implications

To conclude, gender (male and female) differences in NSSI among youth were not found throughout the whole study. Among different types of stressful life events, male students were reported to experience a significantly higher impact of punishment and interpersonal relationship than females. Female students only experienced significantly higher learning pressure than males.

Secondly, several specific types of stressful life events were proved to have a significant impact on youth’s NSSI, proving them as risky factors to youth’s mental health: For male students, the impact of punishment related stressful life events on their NSSI was significant, in other words, the higher impacts of stressful life events related to punishment they experienced, the more NSSI engagement they could have. For female students, stressful life events related to learning pressure, interpersonal relationships and adaption were all proved as significant predictors and risky factors of female youth’s NSSI.

Thirdly, our findings have revealed direct and moderating effects of online social support on NSSI among youth (regardless of their gender) under the pressure of different types of stressful life events, proving that online social support did not influence individual’s NSSI engagement directly, but moderated it significantly (i.e., loss, interpersonal relationships, adaption among males and all types of stressful life events among females) as a protective factor. Especially with the global pandemic still influencing, the uniqueness of online social support (e.g., non - contact, private, etc) [50] could be applied as a long term and effective support source to buffer the negative impact brought by various types of stressful life events, help teenagers to establish a healthy and positive attitude in the current post - COVID world, thereby reducing the prevalence of NSSI or other life - threatening behaviours.

This is the first empirical study to investigate the direct and moderating effects of online social support on NSSI among youth under the pressure of stressful life events in Northwestern China, as well as gender - specific patterns under Chinese culture in these relationships, especially under the afterwave of global pandemic. These findings will inform current study and literature on social support system, stressful life events, and NSSI among youth in China, emphasize the need for longitudinal efforts to explore NSSI and suicidality globally. The results also indicated that middle - high school and university mental health or counselling centers providing necessary support from the internet are the important and crucial resources for students who seek social support from regarding their stressful life experience, NSSI or any other health risk even life - threatening issues, in the context of the current epidemic situation. Meanwhile, cultural competency among practitioners working in middle - high school and university mental health or counselling centers is critical in facilitating social support seeking either from online or offline activities, as well as maximizing positive outcomes for youth who experienced or were just about to experience stressful life events or NSSI in their daily life.

## Data Availability

The data that support the findings of this study are available from School of Psychology, Shaanxi Normal University, but ethical restrictions of Shaanxi Normal University apply to the availability of these data, which contains privacy variables that might affect the growth of adolescents’ mental health and were used under license for the current study, and so are not publicly available. Data are however available from the corresponding author upon reasonable request and with permission of School of Psychology, Shaanxi Normal University.
